# Comparing Ancient DNA Preservation in Petrous Bone and Tooth Cementum

**DOI:** 10.1371/journal.pone.0170940

**Published:** 2017-01-27

**Authors:** Henrik B. Hansen, Peter B. Damgaard, Ashot Margaryan, Jesper Stenderup, Niels Lynnerup, Eske Willerslev, Morten E. Allentoft

**Affiliations:** 1 Centre for GeoGenetics, Natural History Museum, University of Copenhagen, Copenhagen, Denmark; 2 Unit of Forensic Anthropology, Department of Forensic Medicine, University of Copenhagen, Copenhagen, Denmark; 3 Department of Zoology, University of Cambridge, Cambridge, United Kingdom; 4 Wellcome Trust Sanger Institute, Hinxton, Cambridge, United Kingdom; University of Florence, ITALY

## Abstract

Large-scale genomic analyses of ancient human populations have become feasible partly due to refined sampling methods. The inner part of petrous bones and the cementum layer in teeth roots are currently recognized as the best substrates for such research. We present a comparative analysis of DNA preservation in these two substrates obtained from the same human skulls, across a range of different ages and preservation environments. Both substrates display significantly higher endogenous DNA content (average of 16.4% and 40.0% for teeth and petrous bones, respectively) than parietal skull bone (average of 2.2%). Despite sample-to-sample variation, petrous bone overall performs better than tooth cementum (p = 0.001). This difference, however, is driven largely by a cluster of viking skeletons from one particular locality, showing relatively poor molecular tooth preservation (<10% endogenous DNA). In the remaining skeletons there is no systematic difference between the two substrates. A crude preservation (good/bad) applied to each sample prior to DNA-extraction predicted the above/below 10% endogenous DNA threshold in 80% of the cases. Interestingly, we observe signficantly higher levels of cytosine to thymine deamination damage and lower proportions of mitochondrial/nuclear DNA in petrous bone compared to tooth cementum. Lastly, we show that petrous bones from ancient cremated individuals contain no measurable levels of authentic human DNA. Based on these findings we discuss the *pros* and *cons* of sampling the different elements.

## Introduction

With the introduction of next-generation sequencing (NGS), ancient DNA (aDNA) studies on humans have progressed from analyses of a few hundred basepairs of mitochondrial DNA to large-scale population genomic studies [1–3]. The feasibility of such projects ultimately rely on having access to many ancient samples with sufficient biomolecule preservation. In particular, owing to the indiscriminate nature of 'shotgun' sequencing, the proportion of DNA that derives from the target species—the endogenous DNA content—is a crucial factor. Because DNA degrades over time [4], and skeletal tissues are invaded by microbes, the endogenous DNA content is often very low in ancient samples (<1%) making genome-scale analyses impossible or, at best, very expensive [5]. Thus, recent aDNA research on human remains has focused on identifying new suitable substrates [6–9] and optimizing DNA extraction methods [6, 10, 11], thereby increasing the success rate for identifying samples that are suitable for genome-scale analyses. Owing to high levels of endogenous DNA, the inner part of the petrous bone and the cementum layer in teeth roots are currently recognized as the optimal substrates for such research.

The petrous bone, part of the temporal bone, is the hardest and most dense bone in the mammal body [12]. The otic capsule surrounds and protects the sensory organs of the inner ear, collectively known as the vestibulo-cochlear organ. Gamba et al. [8] compared DNA preservation in petrous bones and teeth from seven individuals, revealing that the endogenous DNA content in petrous bone exceeded the teeth by 5.2-fold on average. However, the teeth used in that comparison were not sampled specifically from the cementum layer, which is crucial to optimize the endogenous DNA yield [6, 9]. Pinhasi et al. [7] refined the petrous bone sampling and recent studies have confirmed that this bone preserves aDNA extremely well, even when the samples are from warmer climates like Africa [13], the Near East [14], or Oceania [15].

However, there are only two petrous bones in each skull, and sampling one leaves a visible hole in the inferior part of the skull. For precious ancient skulls this can be problematic. Moreover, the otic capsule is formed by endochondral ossification by week 18 during gestation [16] and strontium isotope ratios in petrous bone can therefore provide information on the geographic location of the child during pregnancy [17], as well as childhood stable isotopic dietary signals [18]. If the entire otic capsule is used for DNA extraction, this information is lost. If sampling involves removal of a large part of the petrous bone, information on sex [19] and childhood disease [20] may be lost as well. Likewise, tooth sampling can be just as damaging, in particular when only one or a few teeth are preserved. In addition to the obvious decrease in exhibitional value caused by removing teeth from a skull, morphological studies of teeth can provide important keys to population affinities [21], and analyses of tooth wear can yield insights into the diet and age of an individual [22, 23]. Strontium isotope analyses of the enamel holds information on the geographic location during childhood [24, 25], and tooth calculus has proven an excellent resource for studying ancient proteomics [26]. We note, however, that morphological and biomolecular analyses of tooth crowns do not have to be compromised by DNA sampling, if only sampling the root e.g., Damgaard et al. [6], and petrous bones can be sampled with careful drilling without affecting the external temporal bone, which is the part mainly used in morphological studies.

Regardless, when doing irreplaceable damage to precious material it is important that the arguments are based on solid scientific evidence, so the *pros* and *cons* can be evaluated with local archaeologists or museum curators before sampling each skeleton. The current literature does not allow for a direct comparison of DNA preservation in petrous bone and tooth cementum, when both these substrates are sampled optimally. To remedy this, we present a comparative analysis of DNA preservation in tooth cementum and petrous bone obtained from ancient human skulls, from across a range of different ages and preservation environments. The samples span four major time periods and locations (Table 1); (i) Bronze Age from Central Asia, (ii) Viking Age from England, (iii) Iron Age and (iv) Historical period from Denmark. We include samples that appear both poorly and well preserved (defined in method section), as well as petrous bones that have been cremated. In prehistorical times cremation was a common funerary practice in many cultures incouding those of the pre-Roman Iron Age in Scandinavia [17]. This ritual has often left the petrous bones as the only surviving biological remains, and therefore the only substrate on which to attempt an aDNA extraction. Although the DNA backbone fragments faster during heating [27], it is possible that the dense structure of the petrous bone could have protected the DNA even under these extreme conditions. We set out to test that.

**Table 1:** Sample overview. An overview of the 34 sampled skeletons included in this study.

Samples	Age	N	Substrates
BA1-BA6	Bronze Age	6	Teeth and petrous bones
IA1-IA7	Iron Age	7	Cremated petrous bones
V1-V11	Viking period	11	Teeth and petrous
H1-H10	Historical	10	Teeth, petrous bones and parietal bone

By analyzing millions of DNA sequences from each sample obtained by NGS 'shotgun' sequencing, we provide a detailed comparative analysis of aDNA preservation in petrous bone, parietal skull bone, and tooth cementum across a variety of localities, time periods, and preservation states. The results yield new important insights, fundamental for making well-informed decisions when sampling ancient humans remains for genomic research.

## Materials and Methods

All laboratory work was performed according to strict aDNA standards [28, 29] in dedicated clean laboratory facilities at Centre for GeoGenetics, Natural History Museum, University of Copenhagen.

### The samples

A total of 34 ancient skeletons were sampled for this project (Table 1). Six were from the Bronze Age period (c. 2100–1800 BC) in Central Asia, 11 were Viking Age individuals from UK (c. 1000 AD), and 10 individuals were from later historical times in Denmark (c. 1650–1850 AD). Petrous bones, teeth, and pieces of parietal skull bone were analysed from the later historical skeletons, whereas we only had petrous bones and teeth available for the Bronze Age and Viking skeletons. Finally, we included seven petrosal bones from cremated Danish Iron Age skeletons (c. 500 BC– 850 AD).

### Targeted sampling

**Fig 1:**
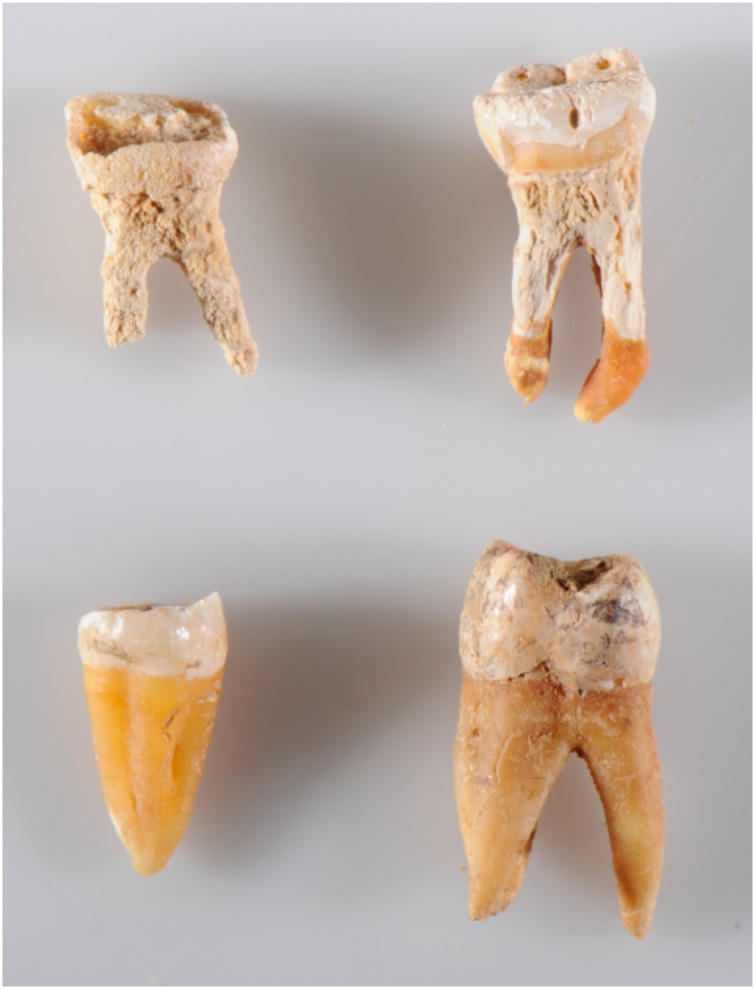
Visual tooth preservation. Examples of poor and good tooth preservation as recorded from simple visual inspection during the sample processing. The upper (poor preservation) teeth display a root with a brittle and "chalky" appearance and the cementum layer is either fragmentary or completely gone. This in contrast to a thick, hard and compact cementum layer observed in the two lower teeth (good preservation).

All tooth samples were cleaned mechanically by gently running a sterile disc drill over the outermost surface. We targeted the cementum in the outer layer of the root by removing as much dentine as possible according to previous guidelines [6]. In poorly preserved samples, where the cementum was absent or partly absent, we still targeted the outermost layer of the roots. The petrous bones were sampled by cutting off the apex part, slice by slice, until the otic capsule was reached. Samples from the same individual (tooth, petrous bone, parietal bone), were always treated in the same batch throughout extraction and library preparation to minimize the effect of potential variation introduced in the laboratory. When processing the samples, a crude visual state of preservation was recorded (Table 2). In the teeth this was defined by the texture and presence/absence of tooth cementum. In particular, the 11 Viking skeletons displayed poor tooth preservation with a more brittle and "chalky" appearance of the roots, and the cementum layer being either fragmentary or completely eroded away (Fig 1). This was in contrast to a thick, hard and compact cementum layer observed in most of the Bronze Age and later Historical samples (Fig 1). Likewise it was recorded when cutting into to otic capsule of the petrous bones if they appeared hard and solid or displayed a more soft and brittle texture (Table 2). Although these are crude binary categories of "well-preserved" and "poorly-preserved", they provided a useful qualitative framework in which to discuss the measures of molecular preservation obtained from the DNA sequencing.

**Table 2:** Endogenous DNA content and visual preservation. A complete overview of the endogenous DNA content and the visual state of preservation (as defined in the method section) for each of the non-cremated samples. Sample names: T = tooth, P = petrous bone, S = parietal (skull) bone. WP, well-preserved. PP, poorly preserved.

Sample	Age	Substrate	Endo. fraction (%)	Visual preservation
H1T	Historical	Tooth	56,1	WP
H1P	Historical	Petrous	54,8	WP
H1S	Historical	Parietal	2,2	WP
H2T	Historical	Tooth	32,5	WP
H2P	Historical	Petrous	53,4	WP
H2S	Historical	Parietal	2,7	WP
H3T	Historical	Tooth	73,7	WP
H3P	Historical	Petrous	41,6	PP
H3S	Historical	Parietal	10,2	WP
H4T	Historical	Tooth	18,0	WP
H4P	Historical	Petrous	34,9	PP
H4S	Historical	Parietal	7,5	PP
H5T	Historical	Tooth	14,2	WP
H5P	Historical	Petrous	7,0	WP
H5S	Historical	Parietal	0,1	PP
H6T	Historical	Tooth	20,7	WP
H6P	Historical	Petrous	4,7	PP
H6S	Historical	Parietal	0,3	PP
H7T	Historical	Tooth	4,7	WP
H7P	Historical	Petrous	56,3	WP
H7S	Historical	Parietal	1,8	PP
H8T	Historical	Tooth	7,5	WP
H8P	Historical	Petrous	43,8	WP
H8S	Historical	Parietal	0,3	WP
H9T	Historical	Tooth	1,3	WP
H9P	Historical	Petrous	43,2	WP
H10T	Historical	Tooth	0,2	WP
H10P	Historical	Petrous	3,5	WP
H10S	Historical	Parietal	0,2	PP
BA1T	Bronze Age	Tooth	3,8	WP
BA1P	Bronze Age	Petrous	0,5	WP
BA2T	Bronze Age	Tooth	52,4	WP
BA2P	Bronze Age	Petrous	32,0	WP
BA3T	Bronze Age	Tooth	0,1	WP
BA3P	Bronze Age	Petrous	0,1	WP
BA4T	Bronze Age	Tooth	7,0	WP
BA4P	Bronze Age	Petrous	5,8	WP
BA5T	Bronze Age	Tooth	61,0	WP
BA5P	Bronze Age	Petrous	9,6	PP
BA6T	Bronze Age	Tooth	1,3	WP
BA6P	Bronze Age	Petrous	4,9	WP
V1T	Viking Age	Tooth	32,0	PP
V1P	Viking Age	Petrous	63,1	WP
V2T	Viking Age	Tooth	3,2	PP
V2P	Viking Age	Petrous	65,3	WP
V3T	Viking Age	Tooth	4,5	PP
V3P	Viking Age	Petrous	66,8	WP
V4T	Viking Age	Tooth	1,3	PP
V4P	Viking Age	Petrous	62,6	WP
V5T	Viking Age	Tooth	1,4	PP
V5P	Viking Age	Petrous	48,9	WP
V6T	Viking Age	Tooth	10,1	PP
V6P	Viking Age	Petrous	63,5	WP
V7T	Viking Age	Tooth	24,9	PP
V7P	Viking Age	Petrous	66,2	WP
V8T	Viking Age	Tooth	3,7	PP
V8P	Viking Age	Petrous	65,6	WP
V9T	Viking Age	Tooth	0,4	PP
V9P	Viking Age	Petrous	51,8	WP
V10T	Viking Age	Tooth	6,7	PP
V10P	Viking Age	Petrous	66,3	WP
V11T	Viking Age	Tooth	0,7	PP
V11P	Viking Age	Petrous	65,3	WP

### DNA extraction, library preparation, and sequencing

The samples were crushed and dissolved using a digestion buffer containing (per ml) 929 μl EDTA, 10 μl TE buffer, 10 μl proteinase K, 50 μl N-laurylsacrosine and 1 μl phenolred. A volume of 2 ml was used for a 15-minute pre-digestion [6] and this was followed by a 24h digestion at 37°C in 4 ml digestion buffer. The DNA was extracted using a silica-in-solution extraction method described in [30], but using the binding buffer from Allentoft et al. [1] which is efficient in binding short DNA fragments. The blunt-ended double-stranded libraries were built using the NEBNext DNA Prep Master Mix Set (New England Biolabs Inc.) with modifications outlined previously [1, 31] and amplified with indexed Illumina-specific adapters prepared as in Meyer and Kircher [32]. The DNA concentration of each amplified library was measured on a Bioanalyzer before pooled approximately equimolarly and 'shotgun' sequenced (100 bp, single read) on an Illumina HiSeq 2500 platform.

### Bioinformatics and DNA authenticity

The data were basecalled using the Illumina software CASAVA 1.8.2 and sequences were de-multiplexed with a requirement of full match of the six nucleotide indexes that were used. The adapter sequences were removed from all reads using AdapterRemoval 1.5.2 [33] followed by a second adaptor trimming using the overlap approach incorporated in Cutadapt [34], retaining reads with a minimal length of 30 bp. The trimmed sequences were then mapped against the human reference genome Hg19, HS Build37.1 using bwa aln with seed length disabled [35]. We removed unmapped reads with samtools and finally removed the duplicate reads from the bam files using the MarkDuplicates function of picard tools (http://picard.sourceforge.net). The endogenous human DNA content was determined as the number of mapped human reads divided by the number of reads retained after trimming. The overall library sequencing efficiency was determined as the number of reads after removing duplicates divided by the total number of reads before trimming.

Coverage on the mitochondrial and nuclear genomes and additional summary statistics were calculated for each sample using Picard Tools (http://broadinstitute.github.io/picard). To confirm the ancient origin of the DNA we determined several key damage parameters with mapDamage2.0 [36] including the C → T mismatch frequency at first position of sequences, the estimated fraction of deaminated cytosines within single stranded overhangs (δs), and the overhang fraction (λ). We estimated the decay constant for each sample using R, by fitting an exponential model to the sequence length distribution on the declining part of the curve [4]. Lastly an estimator of the theoretical average DNA fragment length in each sample was calculated as 1/ *decay constant* [4, 37]

For samples having mitochondrial genomes with >5X coverage, we determined contamination levels using the contamMix software [38] by comparing mapping affinities of each read to the consensus mitogenome of the ancient sample with mapping affinities to 311 mitogenomes worldwide that could be potential contaminants. Consensus sequences were determined using ANGSD [39], using only sites with a minimum depth of 5X, and the 7 bp at the extremities of the reads were disregarded for the contamMix analyses to minimize the biases introduced by DNA damage.

## Results

### Endogenous DNA

**Fig 2:**
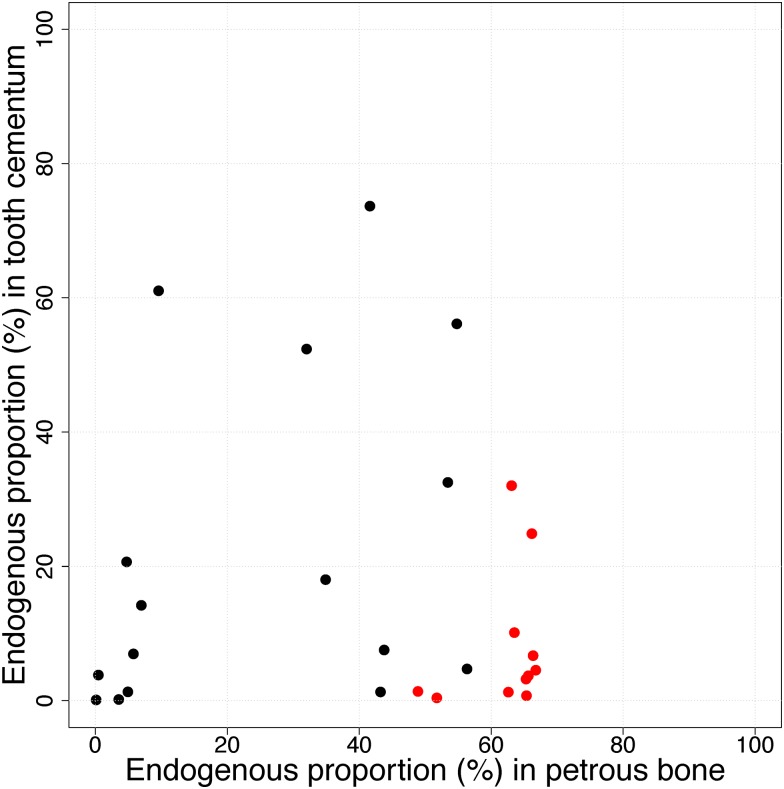
Endogenous DNA content. Each data point represent the relationship between endogenous DNA content in tooth cementum and petrous bones from the same skeleton. Red datapoints represent skeletons in which visual tooth preservation was considered to be poor (see Fig 1).

A total of 1.249.746.005 reads were generated from the 76 samples in this study, avaraging 16.444.026 reads per library (S1 Table). Four million trimmed reads per library have been submitted to the European Nucleotide Archive (http://www.ebi.ac.uk/ena) under accession numbers PRJEB18722/ERP020675. Negligible proportions of sequences were discarded in the bioinformatical trimming and the level of clonality was generally low, albeit with higher levels among samples showing poor molecular preservation (S1 Table). We first compare the endogenous DNA contents in teeth and their paired petrous bone using the entire dataset, regardless of preservation state (Table 2). From this comparison it is clear that petrous bone is overall a better substrate with significantly higher endogenous DNA proportions (paired t-test: t = 3.6, df = 26, p = 0.001) with averages of 16.4% and 40.0% for teeth and petrous bones, respectively. Plotting paired values of endogenous DNA content (in tooth and petrous bone) on a 2-dimensinal plot (Fig 2), we see that the endogenous content in petrous bone is not a good predictor of endogenous content in teeth according to a linear model (coefficient: -0.022, std.err: 0.17, p-value: 0.9). Thus in the overall dataset there is no clear pairwise link between endogenous DNA content in the two substrates. Despite considerable variation it is possible to identify two overall clusters of datapoints representing (1) skeletons with low endogenous content in both substrates, and (2) skeletons with lower endogenous DNA in the tooth than the petrous. There are also outlier skeletons displaying either higher endogenous DNA content in teeth compared to petrous, or high in both substrates (Fig 2).

**Fig 3:**
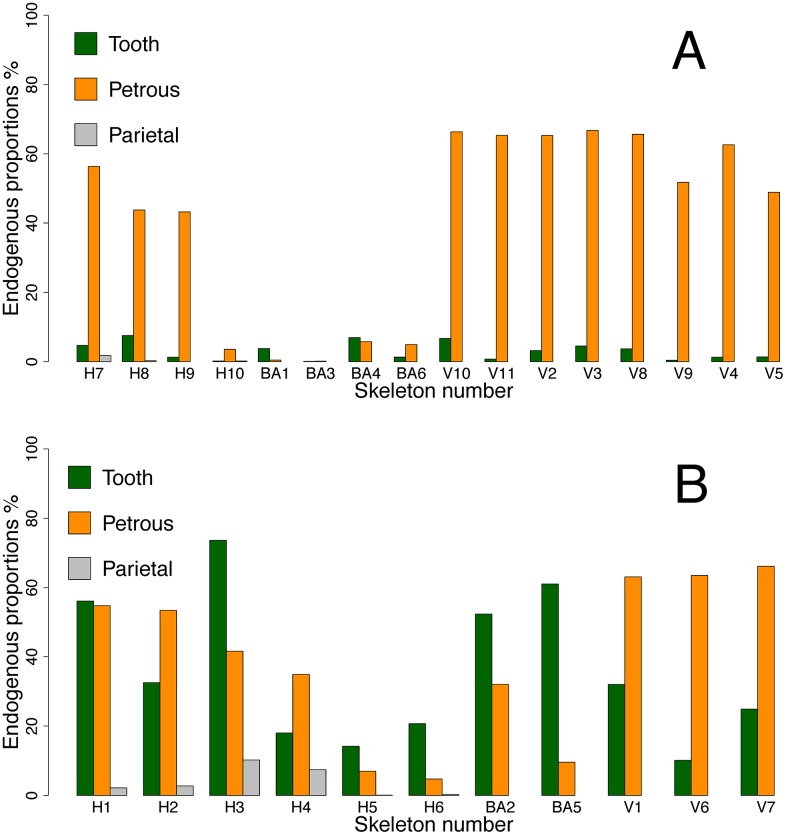
Endogenous DNA content using tooth preservation as reference. Paired endogenous DNA contents in tooth cementum, petrous bone and parietal bone from the same skeleton, using molecular tooth preservation as condition. A) Poor molecular tooth preservation (<10% endogenous DNA), and B) Good molecular tooth preservation (>10% endogenous DNA) respectively.

It is clear that the signal of a better overall molecular preservation in the petrous bones is driven largely by the skeletons in cluster 2, and most of these are Viking Age samples from the same locality. To get a more nuanced picture of the differential DNA preservation in the two substrates, we therefore divided the samples into two simple categories being those with poor molecular preservation (<10% endogenous DNA) and good molecular preservation (>10% endogenous DNA), respectively (Fig 3). There are 11 teeth in the "good" category ranging from 10% to 73%, and the endogenous DNA proportion in the associated petrous bones from the same skeletons range from 4.7% to 66.5% (Fig 3B, Table 2). Amongst these skeletons, five teeth clearly outperformed petrous bone, while in five cases it was the opposite, and in one case the values were nearly identical (Fig 3B, Table 2). We therefore observe no significant difference in average endogenous DNA content between teeth with >10% endogenous content and the respective petrous bones from the same individuals (two-sided t-test for paired samples, t = 0.33, df = 10, p = 0.75). In contrast, the tooth samples with poor molecular preservation (<10%, n = 16), are significantly outperformed by their pairwise associated petrous bones (paired t-test t = 5.5, df = 15, p = 5.39E-05) (Fig 3A, Table 2). Conversely, when using the endogenous DNA content in the petrous bones as condition, the results show that when petrous bones display poor molecular preservation (<10% endogenous DNA), the two substrates are equally poor (paired t-test t = 1.39, df = 7, p-value = 0.21), but when molecular preservation in the petrous bone is good (>10% endogenous DNA) they significantly outperform the teeth (t = 5.7, df = 18, p-value = 2.19E-05).

We also investigated if the simple pre-extraction labels of "good/bad" visual preservation were comparable to the crude good/bad molecular preservation states (above/below 10% endogenous DNA). These measures corresponded in 80% of the cases (Fig 2, Table 2). As an example, the average endogenous DNA content in the visually poor teeth was 8,1% (n = 11) whereas it was 21.2% in the visually good ones (n = 16).

For the nine Historical skeletons, where we also had samples of parietal skull bone, we compared the endogenous DNA content with that of tooth and petrous bone. The endogenous DNA content of the parietal skull bone pieces ranged from 0.1% to 10.2% (Fig 3, Table 2). With an average of 2.8% this is significantly lower than observed in both teeth (t = 2.99, df = 8, p = 0.017) and petrous bones (t = 4.30, df = 8, p = 0.0026) from the same individuals.

Lastly, we investigated whether we could obtain DNA from the petrous bones of ancient cremated skeletons. The seven cremated petrous bones all displayed extremely low levels of endogenous DNA (<0.03%) (S1 Table).

### DNA deamination damage

For the 63 DNA extracts representing non-cremated samples we observe elevated levels of cytosine to thymine mismatches at the 5’ end of the DNA sequences, ranging from 7% in one of the Historical tooth samples to 52% in a Bronze Age petrous bone (average = 26.5%, S1 Table). This is expected for aDNA and confirms the authenticity of the molecules [40]. For this damage parameter we observe a significant difference between the petrous bones and the teeth (two tailed t-tests for paired samples, t = 2.68, df = 26, p = 0.013). When dividing the data into subsets of poor (<10% endogenous) and good (>10% endogenous) molecular preservation we observe that this overall signal is driven by a significant difference between the well-preserved teeth (>10%) and their petrous bone counterparts. In teeth with poor molecular preservation, the C→T damage fraction is not lower than in their associated petrous bones.

By analyzing the distribution of values in different age groups, we find that DNA from teeth of the Historical skeletons display significantly lower C→T values than the older Bronze Age samples (Welch t-test for teeth: t = 3.46, df = 5.98, p = 0.013). This difference is not significant for petrous bone (t = 1.93, df = 5.9, p = 0.10), perhaps indicating that molecular decay in this substrate is less affected by time. These differences are echoed using the DeltaS parameter (S1 Table).

Methylated cytosine at CpG sites decays spontaneously to thymine over time [41] why a higher C→T damage fraction in the petrous bone could potentially be the result of CpG-rich DNA. To investigate this, we performed a two-sided t-test comparing CpG/GpC ratios in tooth and petrous bone from the same individuals. With average CpG/GpC ratios of 0.23 (petrous) and 0.24 (teeth), we observed no difference (t = -1.20, df = 15, p = 0.25) and no outliers, outruling that the observed signals are driven by differential methylation levels (S1 Table).

In the seven cremated petrous bones, the recorded C→T damage fractions at position 1 are very small, ranging from 0.00 to 0.02 (S1 Table). When combined with the extremely low human DNA content (see above), we conclude that we cannot identify any signs of authentic ancient human DNA preserved in these cremated bones.

### DNA fragment length

**Fig 4:**
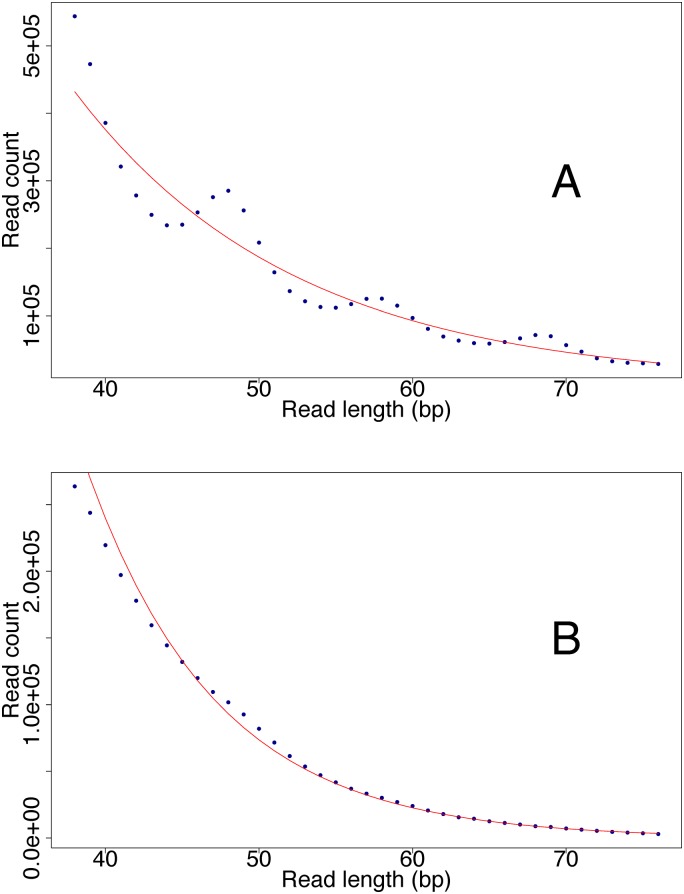
DNA sequence length peridiocity. A signal of 10 bp peridiocity in the length distribution of the human DNA sequences was generally observed more pronounced in the tooth cementum (A) compared to the petrous bone (B). Here this differential signal is shown for skeleton H1 as example. Such 10 bp peridiocity has been observed previously for ancient samples and is believed to reflect preferential cleavage of the DNA strand at positions where the DNA face away from the nucleosome [42].

In teeth, the average length of the reads mapping to the human reference genome ranged from 37.0 bp to 77.1 bp with an overall average of 54.5 bp (S1 Table). In petrous bones from the same individuals it ranged from 39.2 bp to 65.0 bp with an average of 52.9 bp. A two sided paired t-test showed no significant difference between the two substrates (paired t-test: t = 1.19, df = 26, p = 0.24). We correlated the average sequence length with endogenous DNA content and cytosine deamination damage. In this analysis we observe a notable clustering of petrous bones from the viking skeletons (S1 and S2 Figs) standing out with higher endogenous DNA content and longer read lengths than the average sample. Since the observed average read length is influenced by DNA extraction, library preparation, and by removing everything shorter than 30 bp in the bioinformatical trimming step, we also calculated the decay constant from the declining part of the read length distributions, to provide an estimate of the theoretical DNA fragment length in each sample (1/*decay constant*) [4, 37]. There is a tendency of the petrous bone DNA to be more degraded than tooth DNA, with a theoretical average fragment lengths of 19.8 bp and 15.0 bp respectively (S1 Table), but the difference is not significant (t = 1.49, df = 15, p = 0.16). A 10 bp periodicity in the sequence length distribution, reflecting preferential cleavage of the DNA strand where it faces away from the nucleosome [42], is observed in most samples. However, there is a notably difference between the substrates when comparing the ditributions from the 11 teeth with good molecular preservatiton (>10% endogenous DNA), with their petrous bone counterparts. Here the signal is clearly present in the teeth, but not in the petrous bones (Fig 4).

### mtDNA and nuclear DNA

We calculated the ratio of mitochondrial to nuclear genomic coverage in each sample. For the skeletons having teeth of good molecular preservation, the average ratio of mtDNA/nuDNA genomic coverage was 157 in teeth and 79 in the paired petrous bone (S1 Table) and the difference was significant (paired two sided t-test, t = -2.84, df = 15, p = 0.012). In the poorly preserved teeth this skew was even more pronounced, and we observe extreme ratios of mtDNA/nuDNA coverage ratios ranging from 2407 to 13308 (S1 Table).

### Contamination tests

We had sufficient mitochondrial genomic coverage (>5X) from a total of 30 samples to perform the contamination tests (S1 Table). These included 14 teeth and 16 non-cremated petrous bones.

All the estimated contamination levels were very low, ranging from 0.01% to 3.4% (average 0.01%).

## Discussion

### Endogenous DNA

Both the petrous bones and the cementum layer in teeth have previously been demonstrated as excellent substrates for aDNA analyses [6–9] and those observations are echoed in this study. In our comparative setup the petrous bones and tooth cementum samples display on average ~8 times more endogenous DNA than parietal skull bone. In simplified terms this implies that one can on average obtain a genome of 8X coverage for the same price as a 1X genome, when sampling tooth cementum or petrous bone instead of 'regular' bone. Our results therefore consolidate petrous bone and tooth cementum as the best currently known substrates for ancient genomics, and discourage sampling of other skeletal material if teeth or petrous bones are available. Hair samples have previously been reported to yield high quality aDNA [43–45] but ancient hair samples are rare, and failures have also been reported when the samples were preserved under harsh conditions [46].

Based on our results and previous publications, it can be argued that petrous bone is the "safest bet". Under the different preservation conditions covered in this study, petrous bone display high endogenous DNA content more often than tooth cementum. In other words, when ignoring the visual state of preservation, a successful result is more likely to occur from a petrous bone than from a tooth (Fig 3). Importantly however, our results clearly show that petrous bones are not always better than teeth, and this finding nuances previous generalizations about the superiority of petrous bone in aDNA analyses [3]. Given the high site-to-site variation, comparing average values does not do full justice to the complexity within this theme. We countered some of this by splitting the samples into two pools that are discernable in the 2-D visualisation of data: the well-preserved (>10% endogenous DNA) and the poorly preserved (<10% endogenous DNA). In this framework it is clear that teeth with good molecular preservation performed just as well as, and sometimes better than, the petrous bones from the same individuals. In the poor molecular preservation category, however, the teeth showed much lower endogenous DNA content than the petrous bones from the same individuals. This suggests that under harsher preservation conditions, petrous bone is more resistant and/or better protected than tooth cementum, thus preserving biomolecules for a longer time. This likely also explains why petrous bones have proven successful in recent aDNA studies based on material from warmer climates of the Near East [14], Africa [13], and Oceania [15].

From a sampling perspective, these comparisons of DNA preservation are only useful if they can somehow be linked with the visual state of preservation. Therefore, it was reassuring to confirm an 80% correspondance between the simple "good/bad" molecular and visual preservation categories. In the context of tooth vs petrous, it is important to note that when sampling an intact skull, visual inspection of tooth preservation (Fig 1) serves as the only reliable condition to select on, since petrous bone preservation is only clearly revealed upon removing the petrous from the skull and drilling into the vestibulo-cochlear area. Our findings therefore underline that an initial visual inspection of the teeth serve as a highly advisable strategy when sampling skeletal material. Given the *pros* and *cons* outlined in the introduction, we emphasize that when teeth are well-preserved (Fig 1) and have been sampled correctly [6], they can yield just as high, or even higher, endogenous DNA proportions as petrous bone. Conversely, when the tooth root is obviously poorly preserved (no cementum, brittle, 'chalk-like'), it is adviseable to collect the petrous bone instead.

### DNA damage

We generally observed higher C→T damage levels in the petrous bone and were able to reject that this is caused by increased methylation. Another possible explanation could be that tooth cementum is more prone to contamination with modern human DNA (with less C→T damage), in contrast to the inner parts of the petrous bones. However, the contamination estimates rejected this hypothesis, confirming that there is an actual difference in cytosine deamination damage between the two substrates.

Although not significantly different, the average observed mapped sequence length and the theoretical average fragment length (1/*decay constant*) both had a tendency to be shorter in the petrous bones than in the teeth (S1 Table). As C→T deamination damage occurs at the DNA strand breaks, it seems reasonable to assume that an increased fragmentation rate in the petrous DNA is responsible for the slightly shorter fragments and an increased C→T damage rate. We note that the lower C → T mismatch rate in teeth is coupled with the presence of a more pronounced 10 bp periodicity in the sequence length distribution (Fig 4), which has been associated with nucleosome protection of the DNA. This finding could suggest that when the nucleosome is still preserved, it prevents extensive formation of single stranded DNA overhangs, and thereby deamination. However, more analyses are needed to test this.

Histological differences between the two substrates may influence differentially the plethora of oxidative, hydrolytic, and enzymatic DNA decay processes occurring in a dead cell [29], explaininng the molecular differences between the substrates. One should potentially be mindful of these difference when conducting bioinformatical mapping, because reads with more damage, as seen in petrous bone, are less likely to align to a reference sequence than the less damaged DNA from the teeth. Allowing for more DNA damaged reads by extending the edit distance in bwa aln when mapping ancient DNA data indeed increase the number of hits, and importantly, the observed deamination level. However, it also drastically increases mapping processing time and it is for this reason not recommended unless dealing with highly challenging and important samples.

### Mitochondrial vs nuclear DNA

We observe a much higher proportion of mtDNA in the tooth cementum compared to the petrous bone, perhaps suggesting that the tooth cementum is more metabolically active than the petrous bone tissue. Similar results were observed by Adler er al. [9], observing that the cementum layer contained five times more mtDNA than the dentine in ancient teeth. This histologically-related difference in DNA preservation has since been further investigated [47], indicating that the nuclear DNA fragments at a higher pace than the more protected and circular mtDNA. Thus a higher proportion of nuclear DNA is fragmented below the 30 bp cut-off and will be removed in the bioinformatic trimming. The skew in mtDNA/nuclear DNA that we report here is extreme in the poorly preserved teeth with an average mtDNA/nuDNA coverage of nearly 5000, which is c. 20 times more skewed than in the average well-preserved tooth. We suspect that this severe skew is an effect of nuclear DNA degrading much faster than mtDNA under harsher preservation conditions and that this is more pronounced in a tooth than in a petrous bone. This is supported by our data, showing that overall, the mtDNA sequences from teeth are longer than the nuclear DNA fragments (paired t-test, t = 4.42, df = 25, p-value = 1.6E-4) but this is not the case in petrous bone (t = 0.29, df = 24, p-value = 0.77).

We note that because of the higher mtDNA content in the tooth cementum, it will be possible to determine, for example, the mtDNA haplogroup with much less sequencing effort than required for petrous bones. The negative effect on the autosomal sequencing effort is marginal because despite of the skew, mtDNA reads still only take up a small fraction of the total number of mapped reads (max value observed = 0.05% of mapped sequences).

### Cremated petrous bones

We were not able to identify any authentic ancient human DNA in the cremated petrous bones from the Danish Iron Age skeletons. Clearly, the heat during cremation has fragmented the DNA beyond analytical recovery. Admittedly, we only have seven cremated bones in this analyses which are too few to definitively reject this possibility. Furthermore, cremation practices (including the degree of thermal damage) were different between cultures and time periods. However, unless contradicting results emerge in the future, our findings indicate that it is pointless to sample petrous bones from cremated skeletons for the purpose of genomic analyses. In turn, such samples have been shown to be useful in yielding strontium isotope ratios [17].

## Conclusion

We believe that the results presented herein provide aDNA researchers with important new arguments, necessary for making informed decisions during the sampling process. Future research should aim at refining our understanding of DNA decay in space and time. Large-scale studies that manage to correlate aDNA preservation with parameters such as age, average temperature at burial depth, soil pH, storage time and metagenomic profile, should be highly informative—in particular when the effect varies greatly between different sites as seen in this study. Clearly the preservation environment of the Viking Age skeletons has resulted in both poor visual and molecular preservation of the teeth, but not affected the petrous bones, whereas the Bronze Age samples, despite thousands of years older, showed a much better tooth preservation. Here the objective has been of a more practical character, namely to generate empirical data that are easily translated into protocol optimizations. We will therefore end with a couple of simple take home messages that should be useful in that regard. Future refinements of the sampling processes may nuance these statements, but this is the current state of knowledge:

- Tooth cementum and petrous bones are both excellent substrates for ancient genomic research.
- We observe high sample-to-sample variation but overall the petrous bones perform better than the teeth.
- We confirm a link between visual and molecular preservation, and when teeth are well-preserved, they perform on average just as well as the petrous bones.
- Petrous bones display a higher C→T damage rate, and have smaller mtDNA/nucDNA ratios compared to tooth cementum.
- Cremated petrous bones are not suited for genomic analyses.

## Supporting Information

S1 Fig Endogenous DNA content correlated with average length of mapped reads, based on sequencing data from all DNA extracts. Names refer to the sample names listed in Table 2. T = tooth, P = petrous bone, S = parietal (skull) bone.
(PDF)

S2 Fig C→T damage rate at position 1 correlated with average length of mapped reads, based on sequencing data from all DNA extracts. Names refer to the sample names listed in Table 2. T = tooth, P = petrous bone, S = parietal (skull) bone.
(PDF)

S1 Table Complete overview of samples, raw data, and results. (XLS)
