# Supplementary figures and images for: Comparing Ancient DNA Preservation in Petrous Bone and Tooth Cementum
Source: PLoS One. 2017 Jan 27;12(1):e0170940. doi: 10.1371/journal.pone.0170940 (PMC5271384; doi:10.1371/journal.pone.0170940)

Endogenous content %

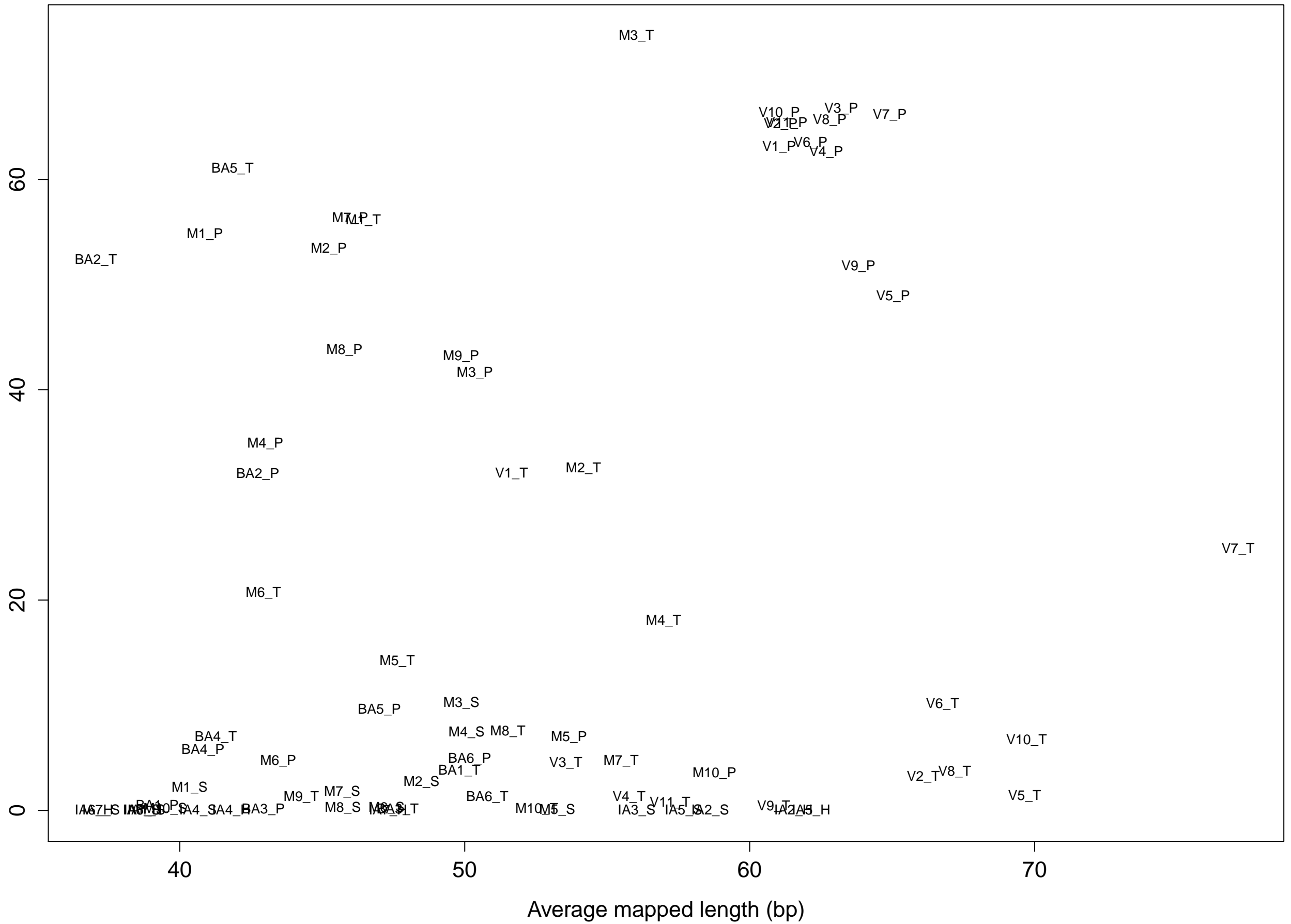

Supplement: S1 Fig — Names refer to the sample names listed in Table 2. T = tooth, P = petrous bone, S = parietal (skull) bone. (PDF) [file pone.0170940.s001.pdf]

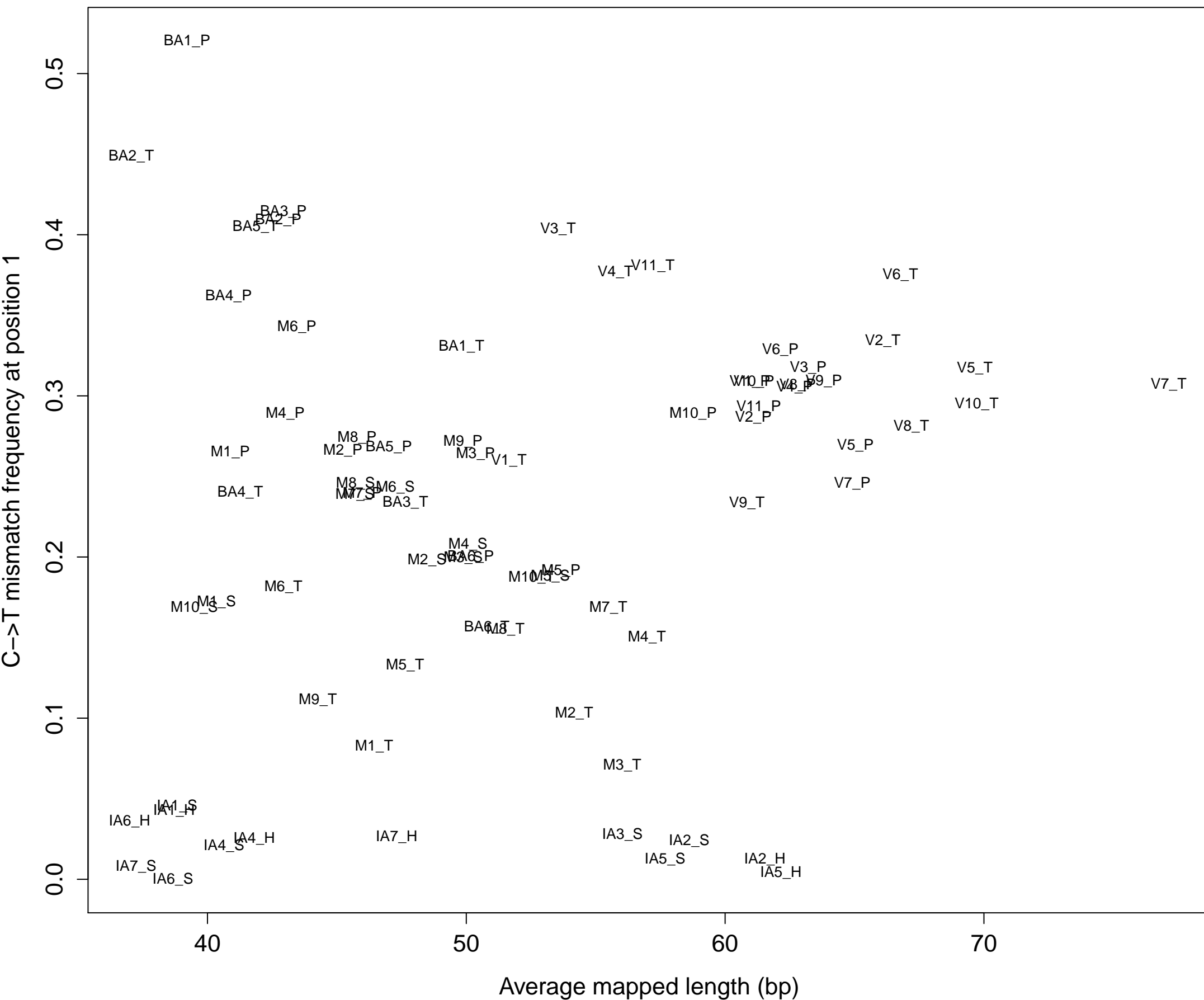

Supplement: S2 Fig — Names refer to the sample names listed in Table 2. T = tooth, P = petrous bone, S = parietal (skull) bone. (PDF) [file pone.0170940.s002.pdf]
